# The 2023 Garrod Lecture: Antimicrobial use and stewardship in primary care: what works, and what next?

**DOI:** 10.1093/jac/dkaf053

**Published:** 2025-04-10

**Authors:** Paul Little

**Affiliations:** Primary Care Research Centre, University of Southampton, Southampton, UK

## Introduction

This article is based on the 2023 Garrod Lecture. In summary, it deals with the behavioural context and the harms of antibiotic prescribing in primary care, outlines simple interventions that would help both prior to and in the consultation, and provides suggestions as to the next steps.

## The problem?

### Behavioural context and evidence for the benefit of antibiotics

Acute infections generate the commonest symptoms experienced in the population. Of those attending primary care each year, 25%–30% have respiratory tract infections (RTIs),^1^ and 10% of women have urinary tract infections (UTIs).^2–4^ Infections are still the biggest causes of sickness and sickness-related work absence (e.g. 30% in 2022 in the UK data from the Office for National Statistics).

People who attend are particularly concerned about symptoms and commonly expect a prescription^5,6^ but the impact of prescribing antibiotics is modest for symptoms.^7–9^ In routine primary care, complications are rare and harms from not prescribing are, in fact, probably minimal;^10^ a typical practice of 7000 people would experience only one extra adverse event per year for a 10% reduction in antibiotics. For uncomplicated UTI, a recent meta-analysis of individual patient data (IPD) suggests that antibiotics do help symptoms and may prevent complications,^11^ and that non-antibiotic strategies increase incomplete recovery and pyelonephritis. However, most of the complications were associated with non-steroidal anti-inflammatory drugs (NSAIDs) so it may be a particular effect of these drugs,^12^ the impact of a placebo being much less. Better non-antibiotic strategies are needed in order to show that the outcome of UTIs is not inferior to that obtained with antibiotics.

### Harms of prescribing in primary care

Prescribing antibiotics creates an inefficient cycle of beliefs and behaviour (people attribute symptom resolution to antibiotics, even if it is not true). In 1997, Little *et al*.^13,14^ showed that offering no antibiotics or delaying antibiotics didn’t result in differences in symptom resolution compared with immediate treatment, but did change believe in antibiotics, and reduced repeated consultations for sore throats subsequently. Observational data also suggest that practices that reduce antibiotic use also reduce consultations going forward.^15^

Anaphylaxis is a rare but potentially devastating harm of antibiotic use (0.3%–2% lifetime incidence, with 25%–40% being due to drugs, mostly penicillin).^16^ Conversely, antibiotic-associated diarrhoea is common—a 17% incidence following antibiotic prescription, but this can be halved to 8% with probiotics.^17^  *Clostridioides difficile*-associated diarrhoea (Goldenberg *et al*.^18^) is also not uncommon—4% with antibiotics, but reduced to 1.5% for people given probiotics. A review using data from shorter versus longer courses of antibiotics has estimated that for every extra day of antibiotics there is a 4% increase in the odds of adverse events, and a 9% increase in the odds of severe adverse events.^19^

### Antimicrobial resistance (AMR) and primary care

AMR has been recognized as a major public health threat for some time by, among others, the WHO (https://www.who.int/health-topics/antimicrobial-resistance), potentially leading to the inability to treat common complications, and making modern surgery and cancer care impossible since they require effective antibiotics.

But, is AMR a problem for primary care? Similar amounts of antimicrobials are used in animals and humans,^20^ with most medical antibiotics being prescribed in primary care [e.g. 73% in England: English Surveillance Programme for Antimicrobial Utilization and Resistance (ESPAUR), 2021]. The next question is whether primary care use really impacts resistance? The ecological study by Goossens *et al*.^21^ shows a clear country-level relationship with prescribing in primary care, and the systematic review by Bell *et al*.^22^ also shows this at an individual level.

The World Bank estimates a 1% annual loss in GDP due to AMR and 5%–7% in low- and middle-income countries (LMICs) by 2050, equating to a cost of around $100–200 trillion dollars.^23^ One of the worrying features of AMR is that is it has not necessarily been shown to be easily reversible; Sundqvist reviewed seven large-scale community interventions, of which three were effective in lowering antibiotic use but did not lower resistance,^24^ and Sundqvist suggests that we are probably overestimating the usefulness of the fitness cost of resistance.

Therefore, alternative strategies to using antibiotics are badly needed for both treatment and prevention. Government investment is also needed due to market failure—since pharmaceutical companies are unlikely to invest the substantial resources to develop new antibiotics with a low rate of return because the drugs are unlikely to be widely used.

## Before the consultation: what works?

Vaccination is clearly central to the prevention of respiratory infections, but for the current article I will concentrate on behavioural interventions that have been developed and trialled in community settings. The three general principles for developing complex interventions that work^25^ comprise: (i) the best evidence and sensitivity to context; (ii) underpinning by behavioural theory; and (iii) prototypes developed iteratively to ensure maximal engagement,^25^ using methods such as the person-based approach (PBA).^26,27^

### Handwashing in the community

A behavioural website to support handwashing was robustly developed using the PBA, then trialled among more than 20 000 participants in the MRC PRIMIT trial.^28^ Over the winter season the intervention demonstrated an important reduction in the incidence of respiratory infections in the index individuals (risk ratio 0.86), a reduction in transmission to family members (risk ratio 0.74), lower antibiotic use (risk ratio 0.83) and fewer gastrointestinal infections (risk ratio 0.84).^28^

### Supporting immune function: nasal sprays, physical activity and stress management

Limited evidence suggests that using common nasal sprays, or improving immune function through increasing physical activity and managing stress, could reduce RTI duration.^29–35^ A much larger trial, the Immune Defence Study has now been published,^36^ studying 13 799 participants with either risk factors for complicated illness or a history of recurrent infections. Compared with usual care, three interventions were assessed: (i) Vicks First Defence nasal spray, which buffers pH to provide an acid environment harmful for viruses, and has a polymer to trap viruses; (ii) saline nasal spray; or (iii) a brief website promoting physical activity and stress management, developed using PBA. In 6 months, compared with usual care, the incidence of infections was reduced by the behavioural website (risk ratio 0.95), and both sprays reduced illness duration (incident rate ratios of 0.82 and 0.81, respectively). All interventions reduced days of more severe symptoms, reduced antibiotic courses (risk ratios of 0.65, 0.69 and 0.74, respectively) and both sprays reduced workdays lost. This suggests that, if widely used, these simple interventions could have a substantial effect in moderating the impact of respiratory viruses in the winter months.

## Alternatives to antibiotics?

### Symptomatic treatment?

NSAIDs probably cause harm in RTIs—increasing prolonged illness/complications, magnifying the cardiovascular risk caused by infections^37–45^—and possibly in UTIs^12^—probably related to the strength/dose of NSAIDs. The increase in prolonged illness, reconsultations and complications results not only in harm to the patient directly but also a cycle of more unnecessary antibiotic use since most people who reconsult get an antibiotic.^46,47^ Occasional NSAID use is probably reasonable, particularly with a delayed antibiotic prescription, since antibiotic use can be reduced without harm, demonstrated in the ATAFUTI trial.^48^ Paracetamol use does not have the disadvantages of NSAIDs,^49^ but sparing use of NSAIDs could be considered. Also worth considering are steroids,^50,51^ for acute, uncomplicated illnesses, and anaesthetic ear drops for otitis.^52^

### Antimicrobial stewardship (AMS)

The systematic review by the Agency for Healthcare Research and Quality (AHRQ)^53^ documented effectiveness for interactive clinic-based participant communication, electronic decision support, and the use of near-patient tests, and a prior review also documented the effectiveness of delayed prescriptions (DPs).^54^

### DPs for antibiotics

DPs (where patients are advised to use antibiotics if the illness is not settling) can substantially reduce antibiotic use, and change beliefs and consultation behaviour as effectively as not prescribing at all.^55–58^ A systematic review highlighted the slightly higher use of antibiotics than a ‘no initial prescription’ approach,^59^ but both achieve low antibiotic use from UK trial data (‘delayed’ 35%; ‘no initial prescription’ 26%^56^). Conversely, a ‘no antibiotic’ strategy may increase both complications (uncommon) and repeat consultations for progressive symptoms (common: approximately 15%–20%), though DPs reduce such repeat consultations by 40%.^47,60^

However, using DPs for *all* consultations is unnecessary and likely to be less acceptable for healthcare professionals (HCPs). For the few individuals at higher risk or who are very unwell, an immediate antibiotic is appropriate; conversely, where HCPs have assessed risks and potential benefit as being low, a ‘no antibiotic’ strategy can be negotiated. Where there is more clinical uncertainty or significant patient pressure, a DP is particularly helpful, allowing rapid antibiotic access should deterioration occur, but sufficient time for most infections to settle. A recent trial^55^ and large cohorts^47,60^ confirm that targeting DPs to those at intermediate risk could improve symptom control, reduce antibiotic use, and may reduce rare complications. However, only one trial for sore throat has assessed targeting DPs using a clinical score.^55^

### Decision aids

Prescribers currently have few practical tools to use in consultations to support effective and safe reductions in prescribing. Our trial of a diagnostic decision aid for sore throat (FeverPAIN) developed with users (patients and prescribers)^55^ reduced antibiotic use and improved symptom control. FeverPAIN is popular—now used several hundred thousand times, and has been endorsed by NICE.

## Where next? Key areas worthy of further research

1. More research in UTI?

Most research on prescribing strategies has been for RTIs. For UTIs, two pragmatic trials have shown that DPs: (i) can reduce antibiotic use;^61^ and (ii) in conjunction with advice to use NSAIDs, can reduce antibiotic use by a further 30%–40%.^48^

2. Larger trials are needed to see how robust these findings are, and we also need a better understanding of how to engage patients in delaying antibiotics for UTI and the appropriate use of NSAIDs. Better implementation of DPs?

The trials to date of DPs have used a brief approach, with the ‘6 Rs’:

- **R**eassurance: immediate antibiotics are unnecessary
- **R**easons to avoid antibiotics: effectiveness/side effects/allergy/AMR
- **R**elief: supporting paracetamol use (limit NSAIDs)
- providing **R**ealistic natural history information
- **R**einforcing the key message: ONLY use prescriptions if not STARTING to settle in the expected time
- **R**escue (safety-netting)

However, DPs are implemented suboptimally in practice, with much shorter delays advised,^47,60^ and poor communication (other simple aspects of the ‘6 Rs’ are often not used), resulting in higher antibiotic use (50%–60% versus trial data 25%–35%).

Large-scale robust implementation needs to ensure that the benefits of DPs can be achieved at scale. Developing patient materials that HCPs can use to efficiently communicate key messages has proved highly acceptable and effective in our previous trials and will help to standardize and optimize implementation of DPs.

3. More widespread use of electronic decision support?

FeverPAIN is popular—now used several hundred thousand times, and has been endorsed by NICE. However, diagnostic or prognostic clinical algorithms for several other infections have also now become available:

- LRTI prognostic predictors in adults (3C^62^) and children (based on the ARTIC-PC data^63^)
- Otitis media (prognostic predictors^64^)
- UTI (UK TARGET diagnostic algorithms based on our UTIS study^61^)
- Sinusitis (we showed that the diagnostic Berg/Carenfelt algorithm reduced the numbers of individuals eligible for antibiotics,^65,66^ and C-reactive protein (CRP) may also be helpful).^67^

None of these has been developed with patient and clinician input for use as decision aids. Although pharmacists currently do not perform many clinical examinations, the decision aids contain mainly information from the clinical history. This can be supplemented by simple measures that pharmacists could use (blood pressure, oxygen saturation) and also the targeted use of rapid point-of-care tests (POCTs): CRP for lower RTI (LRTI) and rapid antigen diagnostic tests (RADTs) for sore throat. Working with GPs, pharmacists and patients, we have now developed a preliminary app incorporating the above decision tools suitable for use in the pharmacy setting (see current development work below).

4. Scalable training interventions to enhance communication in consultations for acute infections?

Very brief internet training to enhance motivation/skills/confidence using written material interactively with patients was effective. GRACE INTRO^68–71^ in reducing antibiotic use in adults, and trials in the Netherlands^72^ and Wales^73^ suggest this approach should also work in children. Using written materials interactively with patients was important in achieving this result.^69–71^ However, the approach needs to be widened: GRACE INTRO only addressed adults and LRTI (not UTI), with little support for targeting of DPs.

5. More research on the use of biomarkers/POCTs?

The above AHRQ review also advocated procalcitonin (PCT), but PCT is poorly validated^74^ and not pragmatic in primary care (at least two tests over several days). The main POCT contender for LRTI is CRP: a systematic review (Verbakel *et al*.^75^), and our trial in COPD^76^ confirmed short-term antibiotic reductions. However, longer-term follow-up (not in Verbakel *et al*.^75^) for the two large trials,^77,78^ which dominated the review,^75^ demonstrated little effect after one season,^77,78^ largely due to GPs not using the tests. The latter trials^77,78^ had desktop analysers so it is possible that simpler lateral flow tests would be easier to use and have a longer-lasting impact in primary care.

6. Better use of pharmacists?

RAPDs to differentiate viral from bacterial aetiology are widely used (e.g. Europe/USA/Scandinavia) and some work in pharmacies in the UK suggests they could also reduce antibiotic prescribing.^79^ There is preliminary evidence that community pharmacy management of acute illness could be both effective and efficient. An assessment of a cohort of patients managed in community pharmacies as part of the Scottish Minor Ailment Service suggests similar health-related outcomes and substantially lower costs with pharmacy consultations for minor ailments compared with GP and Emergency Department management.^80^ A systematic review of such schemes documents both low reconsultation and high symptom-resolution rates, which suggest appropriate management.^81^ The community pharmacy consultation service (CPCS) scheme in England documented 1300 GP practices actively making referrals in 2021 to 3500 community pharmacies, and 90% of episodes were completed by the pharmacist as a single episode of care, with only 15% being signposted to the GP. Finally, NHS England has commissioned a national ‘pharmacy first’ service, which commenced in early 2024, covering the dispensing of antibiotics for uncomplicated UTIs, impetigo, infected insect bites, sore throat using the FeverPAIN algorithm, otitis and sinusitis. The proposed scheme is still somewhat limited in range (in particular, one of the commonest presentations, namely LRTI, is not included); neither decision-making tools nor POCTs are included (as recommended by the National Action Plan), and whether the scheme is safe and effective is as yet unclear.

### Conclusions

The use of antibiotics and prescribing behaviours represent complex interactions so robust behavioural methods are needed in designing interventions to address them if there is to be any chance of containing AMR. Simple interventions aimed at people prior to the consultation, and developed using the PBA, have been shown to be successful, particularly handwashing and also the use of nasal sprays very early in the illness. Interventions likely to work in the consultation to improve AMS are enhanced communication, delayed prescriptions and clinical decision aids. Further research is needed to better engage patients and prescribers to extend the reach of existing interventions, including further exploring the role of pharmacists.
